# Pleural empyema associated with alveolar‐pleural fistulas in severe acute respiratory syndrome coronavirus 2

**DOI:** 10.1002/ccr3.4262

**Published:** 2021-06-22

**Authors:** Duilio Divisi, Gino Zaccagna, Chiara Angeletti, Elena Cicerone, Andrea De Vico, Riccardo Moretti, Giovanna Imbriglio, Nadia Carbuglia, Gina Rosaria Quaglione, Sofia Chiatamone Ranieri, Roberto Crisci

**Affiliations:** ^1^ Department of MeSVA Thoracic Surgery Unit Giuseppe Mazzini” Hospital University of L’Aquila Teramo Italy; ^2^ Intensive Care Unit Giuseppe Mazzini” Hospital Teramo Italy; ^3^ Division of Pathology and Histology “Giuseppe Mazzini” Hospital Teramo Italy; ^4^ Department of Laboratory Medicine Clinical Pathology and Microbiology Laboratory Giuseppe Mazzini” Hospital Teramo Italy

**Keywords:** alveolar‐pleural fistula, COVID‐19 infection, empyema, prolonged air leaks, surgical treatment

## Abstract

Surgery in COVID‐19 disease complicated by APF represents the last life‐saving treatment option. The choice of the therapeutic period to indicate this approach is fundamental. In fact, the clinical stability of patient is necessary in order to allow single‐lung ventilation and to minimize postoperative sequelae.

## Graphical abstract

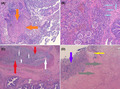

## 1 INTRODUCTION

We describe a SARS‐CoV‐2 characterized by pneumonia, alveolar‐pleural fistulas, and empyema. Patient, initially treated with noninvasive ventilation, required an orotracheal intubation due to the worsening of the clinical conditions. Despite medical treatment with empiric antibiotics, hydroxychloroquine, low molecular weight heparin, glucocorticoids, and monoclonal antibody, patient displayed a right pneumothorax on the thirteenth day, needing pleural drainage. The persistent air leaks and empyema, in spite of targeted antibiotics, required surgical approach, with empyemectomy and suture of fistulas. In this case report, we evaluate the alternation, also chronologically, of the different therapeutic moments. This management allowed the survival of patient, in spontaneous breathing with tracheotomy, although the wide destruction of the lung parenchyma architecture.

Coronavirus disease completely subverts the lung structure through parenchymal necrosis due to widespread vascular thrombosis,^1^ a wide inflammatory reaction and a squamous metaplasia linked to trauma on the mucous membrane of the bronchioles. The diffusion and adaptability of the coronavirus to the human population seem to be linked to the action of furin,^2^, ^3^ which cleaves the S protein into two subunits: S1 responsible for recognizing the angiotensin‐converting enzyme‐2 (ACE2) receptor and S2 responsible for the passage of the virus inside cells. In such a clinical‐pathological situation, the development of empyema sustained by alveolar‐pleural fistulas reduces the chance of treatment and survival of patients.

## 2 CASE REPORT

**FIGURE 1:**
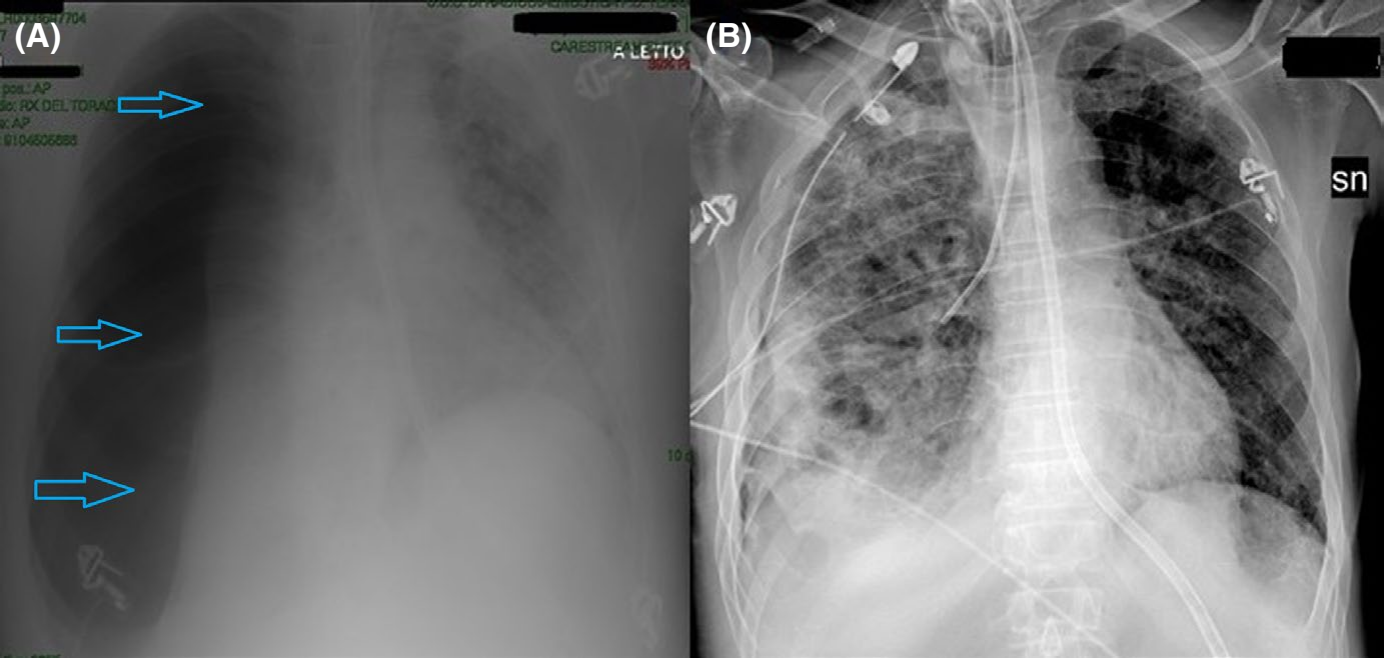
A, Large right pneumothorax (light blue arrows), associated with massive collapse of the homolateral lung; B, Parenchymal re‐expansion after pleural drainage insertion

**FIGURE 2:**
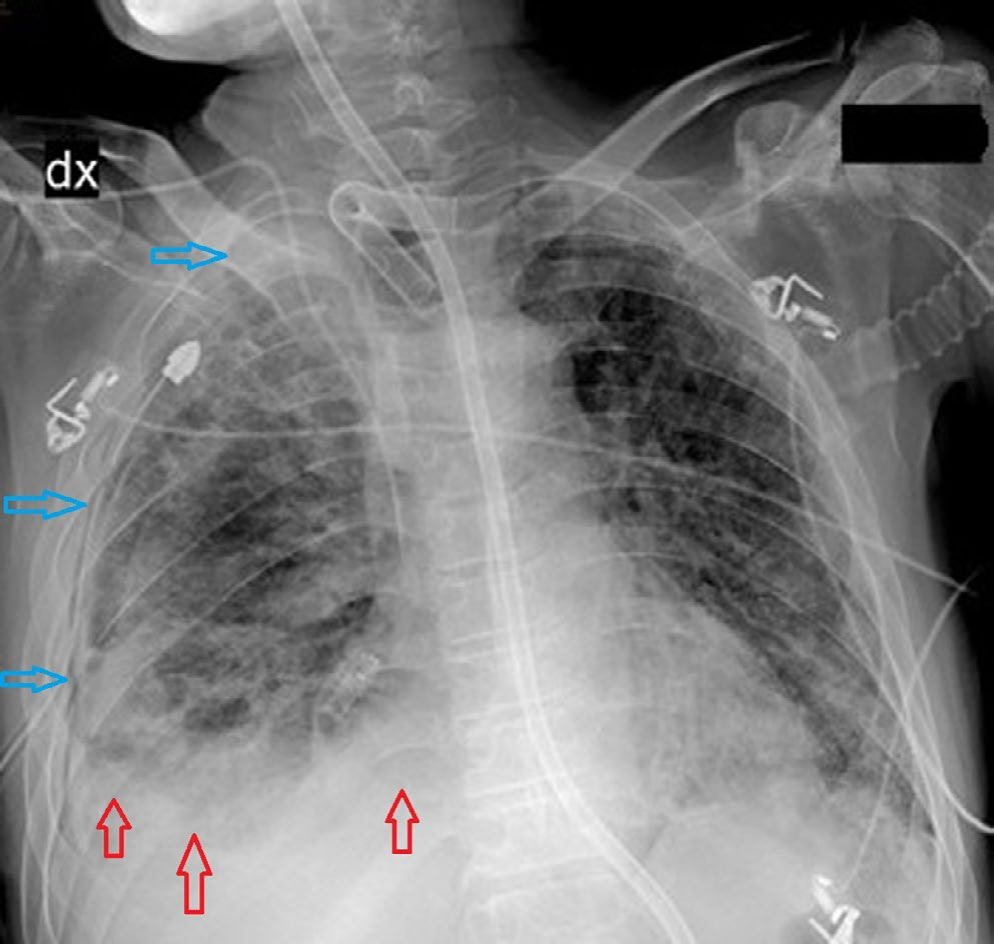
Persistent right pneumothorax (light blue arrows) associated with pleural space infection (red arrows)

**FIGURE 3:**
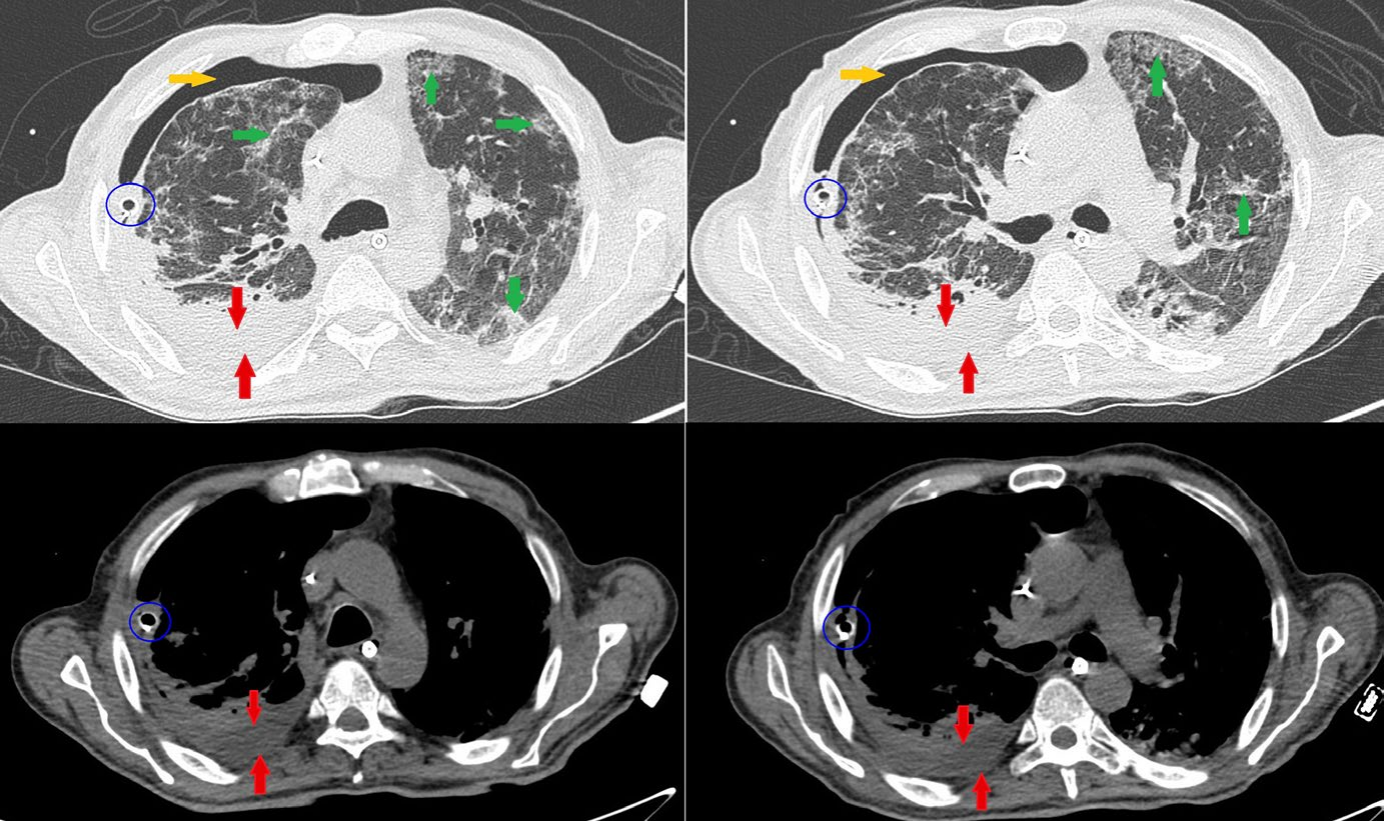
Apical and basal right pneumothorax (yellow arrows) with collapse and atelectasis of the pulmonary parenchyma. Pleural drainage is clearly visible (blue ring). Some air cavitations (orange arrows) are recognized in the lower lobe. These show serpiginous morphology as bronchiectasis and lobulated morphology as pneumatocele. Furthermore, there is a pleural effusion (red arrows) with hyperdense and irregular aspects as corpuscular component

**FIGURE 4:**
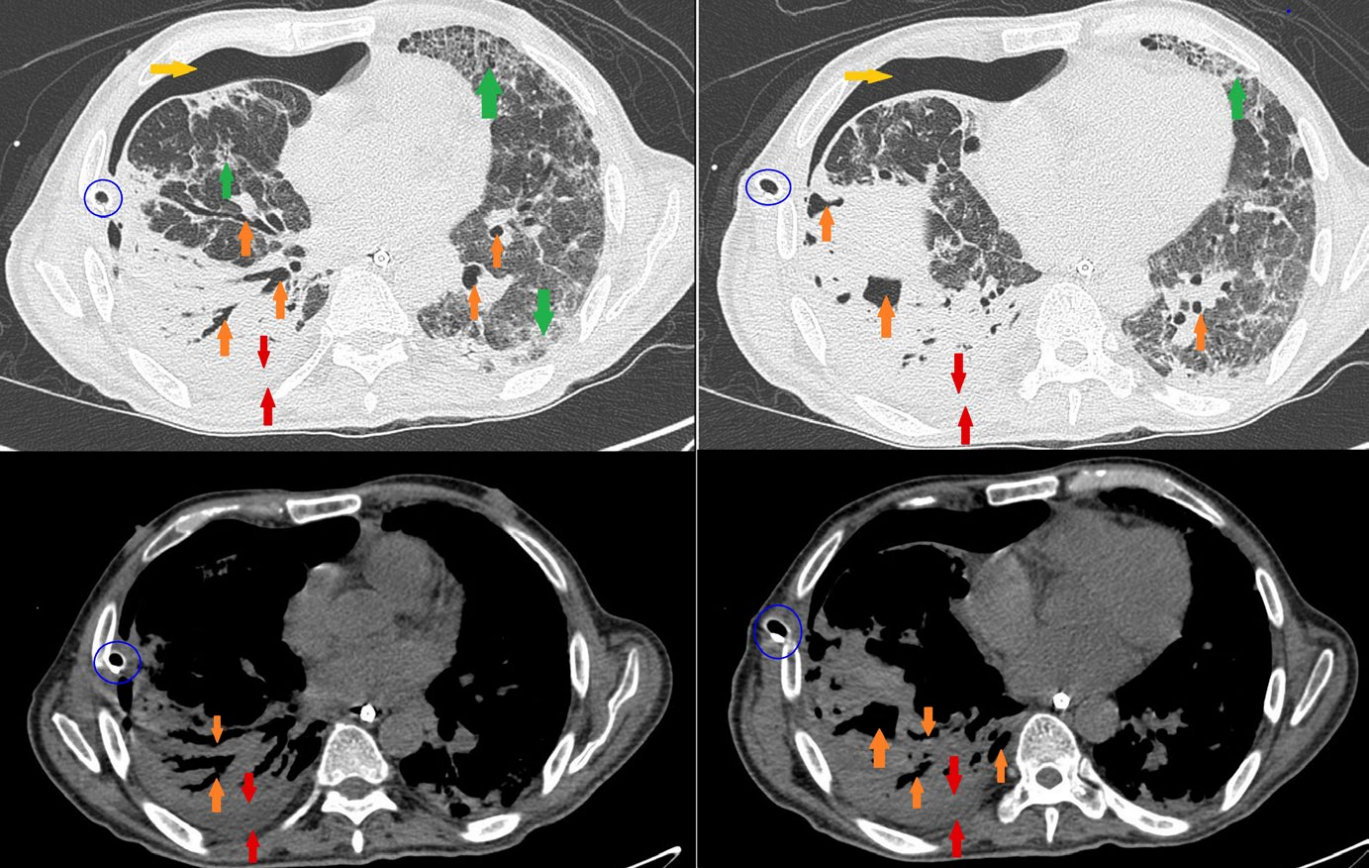
Bilaterally, diffused ground‐glass (green arrows) and interstitial lung disease with an increase in the thickness of the septa are recognized, associated with a basal parenchymal consolidation of the left lower lobe. Moreover, paraseptal emphysema changes are also recognized

**FIGURE 5:**
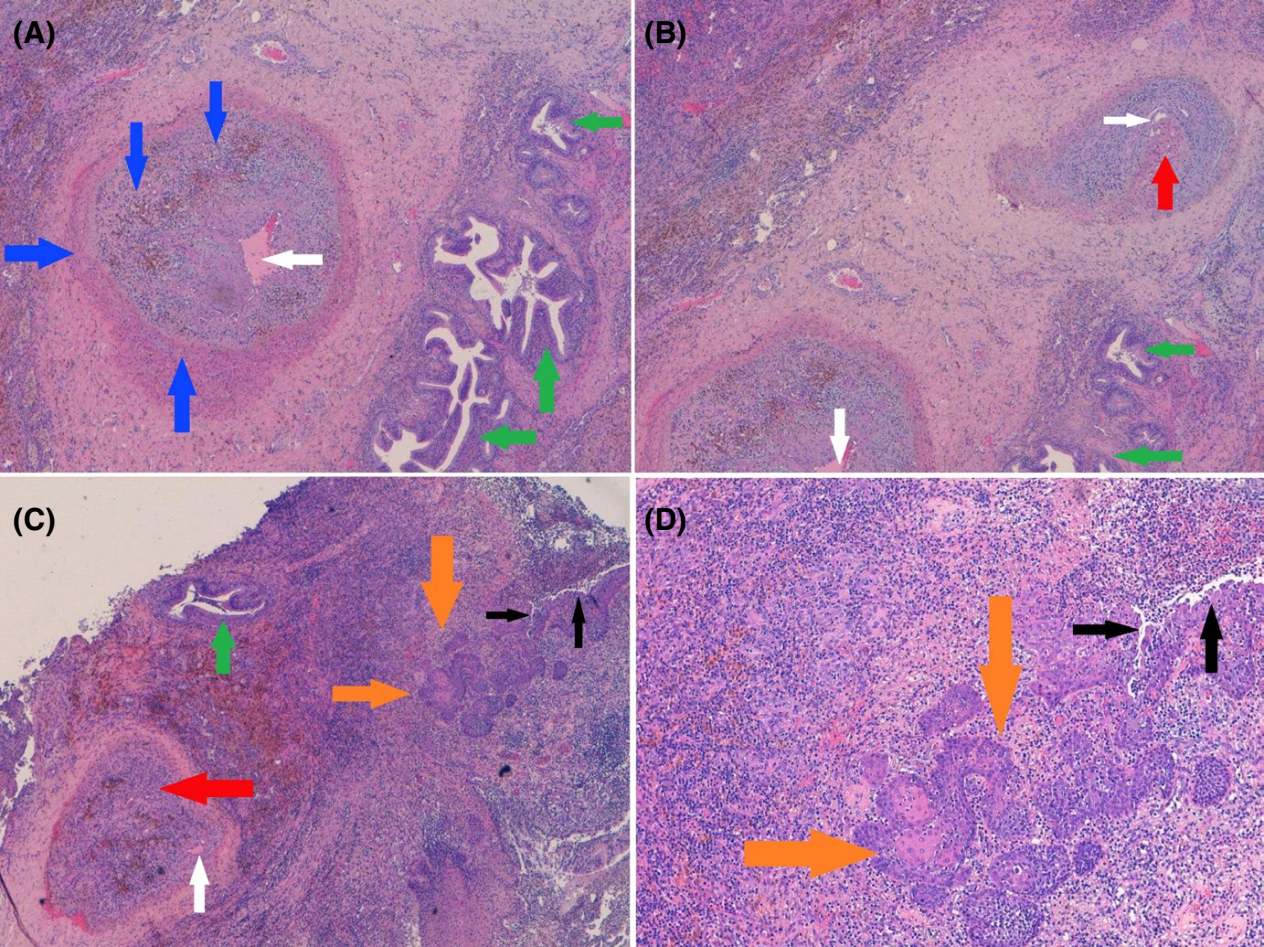
A, Arteriole occluded with micro‐channeling aspect (white arrow), intimal hyperplasia, and siderophages (blue arrows). Chronic peribronchiolar inflammation (green arrows) coexists; B, Thrombosed arterioles (red arrow) associated with an intense inflammatory reaction (green arrows) and micro‐channeling aspects (white arrows); C, Subpleural pulmonary parenchyma with intense inflammation (green arrow). The bronchiole shows fistula (black arrows) and extensive squamous metaplasia (orange arrows). On the side, a thrombosed artery (red arrow) due to intimal hyperplasia with vascular micro‐recanalization (white arrow) is observed; D, Alveolar‐pleural fistula (black arrows), with squamous metaplasia (orange arrows) of bronchial epithelium

**FIGURE 6:**
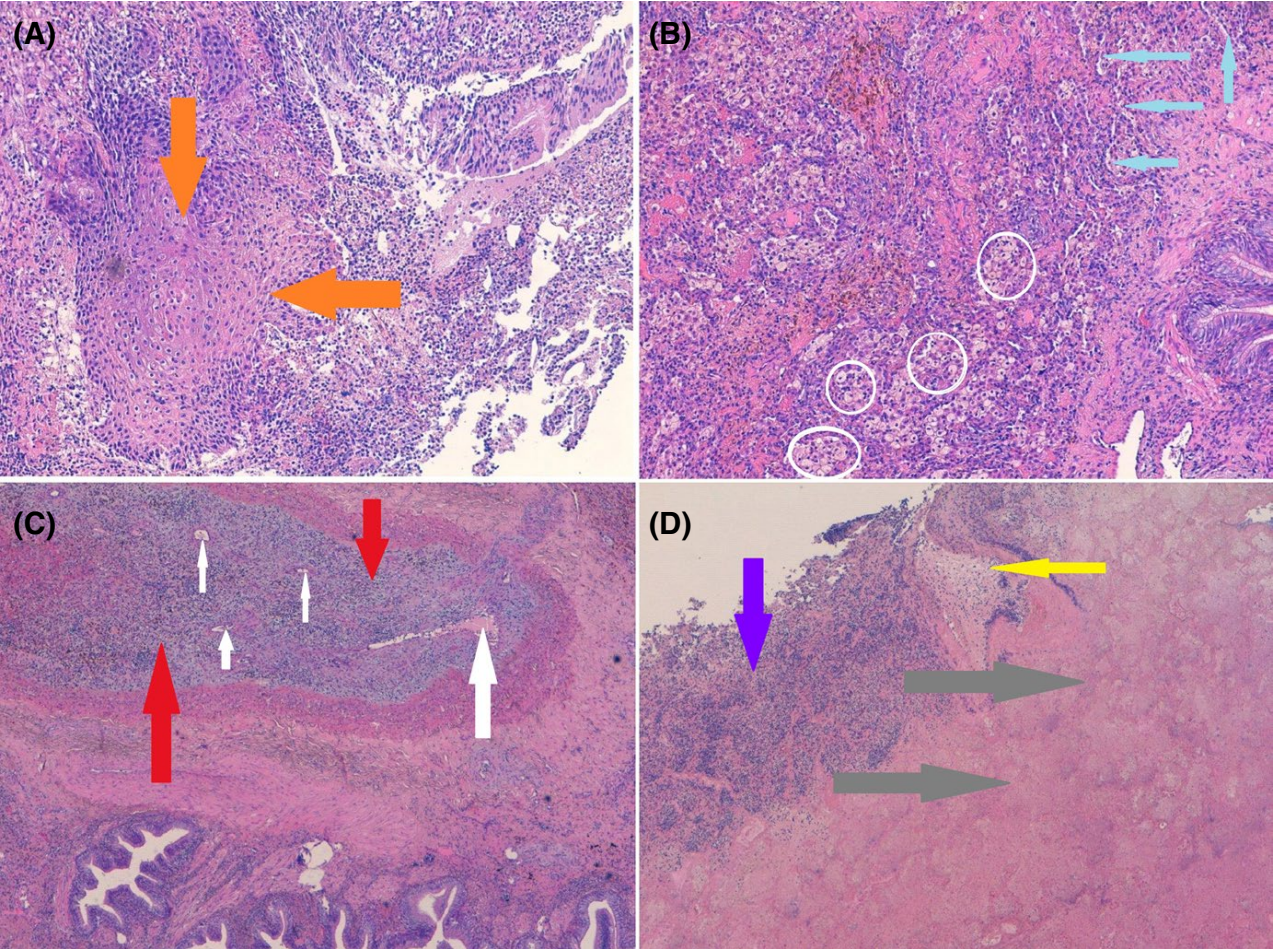
A, The bronchiole with squamous metaplasia (orange arrows); B, Thickened lung interstitium, with inflammatory elements and macrophages (white rings) that have phagocytized hemosiderin (siderophages, outcome of hemorrhage). Alveoli collapsed (light blue arrows) with endoalveolar foamy macrophages are also noted; C, Thrombosed arteriole (red arrows) in which a minimal vascular lumen (white arrows) can be appreciated; D, Pulmonary parenchyma with extensive necrosis (gray arrows), surrounding inflammatory lymphocytes and infiltration of granulocyte neutrophils (blue arrow). Residual vascular structures (yellow arrow) with wall necrosis are also observed

A fifty‐two‐year‐old man, showing fever and dyspnea for 4 days, underwent nasopharyngeal swab, which allowed diagnosis of the novel coronavirus disease 2019 (COVID‐19). The worsening of breathing initially required hospitalization and noninvasive ventilation; subsequently, the patient's clinical conditions necessitated an orotracheal intubation associated with invasive mechanical ventilation (tidal volumes = 6 mL/Kg predicted body weight), positive end‐expiratory pressure (PEEP ≤12 cm H_2_O), and drive pressure ≤12 cm H_2_O. Medical treatment consisted of oral hydroxychloroquine 200 mg twice a day, intravenous (iv) piperacillin/tazobactam 4.5 g three times a day, subcutaneous enoxaparin 6000 UI once a day, iv dexamethasone 20 mg once a day, iv noradrenaline 0.1 mcg/Kg/min, and iv tocilizumab 8 mg/Kg on 3rd day of hospitalization and 12 hours following the first administration. Furthermore, patient was treated with neuromuscular agents (rocuronium) and prono‐supination position for 12 hours a day. A right pneumothorax (Pnx), needing chest drainage 28 French (Fr) linked to digital system, was displayed on 13th day (Figure 1). The air leaks associated with the inability to properly ventilate the patient required the placement of endobronchial obturator into the intermediate bronchus. Percutaneous tracheotomy was carried out on 15th day. Chest X‐ray at 17th day displayed a right pleural effusion (Figure 2); pleural fluid culture revealed a pseudomonas aeruginosa. Such infection was treated with targeted antibiotics (meropenem three times a day and gentamicin 240 mg per day). RT‐PCR was tested negative for SARS‐CoV‐2 in the bronchoalveolar‐lavage fluid at 19th day and 21st day, which also confirmed the pseudomonas aeruginosa infection related to empyema (Figures 3 and 4). Empyemectomy was carried out at 31st day, after stabilization of hemodynamic and respiratory parameters, by muscle‐sparing axillary minithoracotomy (MSAM). Three alveolar‐pleural fistulas (2 in the lower lobe and 1 in the middle lobe) were sutured by black silk separate stitches two zeros and two 32 French pleural drainages were placed. Endobronchial blocker was left in place to protect the sutures, and it was removed on the 4th postoperative day while pleural drainages were withdrawn on the 7th. On the 20th day, patient was discharged and transferred to rehabilitation hospital in spontaneous breathing (O_2_, 2 l/min with tracheotomy) and normocapnia, with respiratory rate 18/minute and PO_2_/FiO_2_ > 350. The clinical history was summarized in Table 1 and in Table 2. Histologic evaluation (Figures 5 and 6) showed pulmonary parenchyma with fibrosis and blood extravasations, associated with: (a) fistulas between terminal bronchioles and visceral pleura; (b) squamous metaplasia (positive for cytokeratin 34betaE12 and cytokeratin 5/6, showing proliferation index Ki‐67/MIB1 lower than 1%); (c) arterioles thrombosed, with intimal obliterating hyperplasia and multiple micro‐channeling aspects. The follow‐up after 1 year displayed the cardio‐respiratory recovery, with resumption of daily activities.

**TABLE 1:** Clinical, comorbidity, infective, antibiotic therapy, radiological, and outcome

Age/sex	Risk Factors	Previous ventilation	COVID‐19 Therapy	SOFAs	Superinfection Pathogen	Positive Samples	ABT/days	Involved lobes	Complication/Interventions	Weaning/Outcome
52/M	no	NIV (>7days)	Tocilizumab Hydroxychloroquine	9	Pseudomonas Aeruginosa	BAL Pleural fluid	Piperacillin‐Tazobactam/15. Meropenem +Gentamicin/18	LLL	PNX/Chest tubes APFs/Bronchial blocker Empyema/Thoracotomy‐Empyemectomy, APFs Sutures	51d/Hospital discharge

**TABLE 2:** Laboratory, blood gas analysis, and cardiac parameters during ICU stay

	*ICU* *Admission*	*PNX*	*Pre* *thoracotomy*	*Post* *thoracotomy*	*Hospital discharge*
*WBC count (10^9^/L)*	15.67	17.55	8.58	11.65	6.02
*Nr Neutrophilis (10^9^/L)*	13.68	15.20	5.53	9.87	3.35
*Nr Lymphocytes (10^9^/L)*	0.71	0.9	1.87	0.96	1.99
*Neutrophilis to Lymphocytes ratio*	19.2	16.8	2.9	10	1.93
*LDH (UI/L)*	561	735	500	590	<400
*D‐Dimer (mg/L)*	1.75	5.42	1.23	1.55	<1
*CRP (mg/L)*	270.37	43.09	68.79	62.85	42
*IL‐6 (pg/mL)*	106.6	3618	18.14	38.74	5
*PCT (ng/mL)*	2.53	1.72	0.18	1.06	0.08
*pH*	7.2	7.36	7.4	7.03	7.4
*pCO_2_ (mmHg)*	75	68	48	68	38
*PaO_2_/FiO_2_ ratio/FiO_2_*	108/100%	80/100%	261/60%	204/80%	>300/21%
*SO_2_ (%)*	91^b^	90^b^	100^b^	100^b^	100
*Vasopressor*	‐	Nora 0,2 mcg/Kg/min	‐	Nora 0,2 mcg/Kg/min	
*MAP*	>70	<65	>70	<65	>70
*HR (bpm)*	88	125 (AF)	98	110	80
*Urine output (ml/h)*	200^a^	150^a^	220^a^	170^a^	150
*Lactate (mmHg)*	0.6	1.6	0.8	24	0.7

^a^ under diuretic therapy.

^b^ mechanical ventilation.

## 3 DISCUSSION

Our experience allows different key points to discuss. Pneumothorax (pnx) was due to thrombosis of interstitial blood vessels, followed by parenchymal necrosis and loss of tissue elasticity. Singhania et al^4^ trace the hypercoagulability in COVID‐19 to the simultaneous involvement of all components of the Virchow's triad (endothelial injury, stasis, hypercoagulable state). This coagulopathy explains emphysema, pulmonary pseudocysts as pneumatoceles and continuous air leaks, exacerbating acute respiratory distress syndrome (ARDS) in severe CoV‐2 pneumonia. Therefore, the pneumothorax and alveolar‐pleural fistulas (APFs) were not related to the barotrauma in our experience as shown by Yang et al^5^ in only one patient with pnx (2%) but rather to the severe fragility of the lung due to the destruction of the parenchymal architecture. The immunocompromised state may have contributed to pseudomonas aeruginosa infection but not to development of fistulas linked to alveolar damage as already described in literature.^6^, ^7^ Furthermore, we have never found the growth of mycosis in cultures as displayed by Placik et al^8^ Such considerations justified the percutaneous insertion of a large caliber chest tube (at least 28 Fr), as its function is not only of decompression but also of constant air drainage in order to ensure an adequate lung recovery. Obviously, the thoracostomy tube placement must be carried out by an experienced doctor or surgeon, assisted only by a staff member as recommended by the AAST.^9^ We used the closed digital chest drain system as it reduces the aerosolization of virus and the exposure of the team, although an alternative method (HEPA filter) has been proposed by Carvalho et al^10^ Concerning the treatment of APF, as it was a Grade C air leak according to Cerfolio classification ^11^ associated with empyema, we opted for a temporary endobronchial blocker in order to stabilize ventilation followed by surgical intervention. In fact, the pleural cavity cleaning and decortication to avoid persistence of infection as well as the suture in layers with separate stitches of APFs are mandatory. The use of staplers appears not to be indicated on a fibrotic, necrotic, and infected parenchyma, as it could not ensure the perfect seal but determine further lacerations of the lung. The last aspect to be evaluated concerns the histological alteration of the pulmonary parenchyma, which creates doubts about the restoration of tissue integrity. Histologically, the SARS‐CoV‐2 infection associated with an acute respiratory distress syndrome (ARDS) is characterized by the vascular and respiratory epithelium damages. The angiotensin‐converting enzyme‐2 (ACE2) receptors, also widely expressed in the endothelial cells, allow the virus to determine a widespread endotheliitis,^1^, ^4^ with hyperplasia and subsequent thrombosis. Ackermann et al^12^ described the same lesions affecting lungs, kidneys, heart, and liver in the autopsy of patients who died from COVID‐19. In addition, the binding of coronavirus to ACE2 receptors and the angiotensin II can directly damage pneumocytes by activation of immune cells system,^13^ reducing the production of surfactant and lung elasticity with development of fibrosis. The cytokine storm was highlighted in our experience by the dosage of Interleukin six between 523.9 pg/mL and 3618 pg/mL in our experience. This may explain the pulmonary edema, interstitial inflammatory infiltrates with lymphocytes predominance, hyaline membrane formation (indicating an ARDS) but it is also related to squamous metaplasia that has already been noticed in deceased patients ^14^ and we have found in our live patient. These histopathologic findings occur through two distinctive manifestations^15^: an exudative early phase and a proliferative and fibrotic phase. The bronchial response to stress or irritation due to APF, as we have observed, was formed on disruption of airway architecture, making the regression of squamous metaplasia extremely difficult. This aspect was also confirmed by immunohistochemistry which accounts for the alveolar damage spread in COVID‐19 infection. In conclusion, it is evident that the attack on the vessels and the airway are the two key moments of the SARS‐CoV‐2 infection with ARDS, causing an impairment of both perfusion and ventilation. In fact, the lesions found in the lungs consisted of the destruction of the pulmonary parenchyma. Surgery becomes necessary in case of fistulas, whether or not associated with infection of the pleural cavity. The indication for surgical approach at the time of greater clinical stability facilitated the functional recovery of patient.

## CONFLICTS OF INTEREST

The authors have no conflicts of interest to declare.

## AUTHOR CONTRIBUTIONS

(I) Divisi D: involved in conception, and provision of study materials or patients; (II) None: involved in administrative support; (III) Divisi D, Angeletti C, and Cicerone E: collected and assembled the data; (IV) Divisi D, Zaccagna G, and De Vico A: involved in data analysis and interpretation; (V) All authors wrote and finally approved the manuscript.

## ETHICAL APPROVAL

The authors are accountable for all aspects of the work in ensuring that questions related to the accuracy or integrity of any part of the work are appropriately investigated and resolved.
